# Spatial analysis of schizotypal personality traits in Chinese male youths: evidence from a GIS-based analysis of Sichuan

**DOI:** 10.1186/1752-4458-8-3

**Published:** 2014-01-15

**Authors:** Jiaxi Zhang, Wei Wang, Zhijun Tan, Qing Wu, Wei Xiao, Lei Shang, Yan Zhang, Jiaxi Peng, Danmin Miao

**Affiliations:** 1 Department of Psychology, Fourth Military Medical University, Chang Le Western Street No.169, Xi’an, Shannxi, China; 2 Department of Statistics, Fourth Military Medical University, Xi’an, China; 3 Foreign Language Teaching and Researching Office of Basic Education Department, Chongqing Communication Institute, Chongqing, China

**Keywords:** Schizotypal personality, Spatial distribution, GIS, Male youth

## Abstract

**Background:**

Schizotypal personality traits are associated with schizophrenia spectrum disorders, stating that schizotypal traits may represent a “prodrome” or other developmental precursor of schizophrenia. Genetic and environmental factors both play importanxt roles in the development of schizotypal traits. Different levels of schizotypal traits across regions may be indicative of similar differences in the incidence of schizophrenia.

**Aim:**

The present study identifying where in a given region, schizotypal personality traits are more or less level of schizotypal personality scores in Chinese male youth of Sichuan province. Not only for research purposes but also for the evaluation of new draft and allocation policy initiatives intended to aid recruitment of mental health employees.

**Methods:**

Data from the Psychological Selection Systems for Chinese Recruits, a mental health screening system used in China, collected in 2011 (67,558 copies) were used to map spatial distribution of schizotypal personality traits using geostatistics and geographic information system (GIS) techniques. Correlation analyses were conducted to explore the effects of years of education and illiterate rate on schizotypal personality traits.

**Results:**

Maps for three schizotypal personality clinical scales (dissociative, Dit; neurotic, Net and sensitive, Set) showed similar geographical trends. The highest T scores were distributed mainly in the eastern and northern counties of Sichuan, with scores decreasing successively from east to west, with the eastern counties generally showing higher scores. Correlation analysis showed that t-scores of Set were negatively correlated with years of education, whereas t-scores of Net were negatively correlated with illiteracy rate.

**Conclusions:**

Schizotypal personality traits in male youth showed specific geographical trends in Sichuan province, providing some evidence that kriging based on GIS can be used to geographically localize genetic and environmental factors associated with schizotypal personality traits. This approach could be used to help allocate public health resources to specific areas and could also have personnel selection applications.

## Background

As an important endophenotype for schizophrenia, schizotypal personality is characterized by odd behavior and attenuated forms of the features seen in schizophrenia, albeit without manifestation of overt and sustained psychosis [1,2]. Studies show that schizotypal personality has a relatively consistent dimensional structure in the general population, with the majority of evidence supporting three symptom clusters: Cognitive-Perceptual deficits (comprised of Ideas of Reference, Magical Thinking, Unusual Perceptual Experiences, and Paranoid Ideation), Interpersonal-Affective deficits (Social Anxiety, No Close Friends, Blunted Affect, Paranoid Ideation), and (3) Disorganized behavior (Odd Behavior, Odd speech) [3-7]. Xiao [8] identified 140 highly specific behavioral traits that characterize schizotypal personality in Chinese samples, in a longitudinal study of young schizophrenics and schizotypal personality disordered patients. Factor analysis was used to reduce this set of behavioral traits to three broad dimensions (dissociative, Dit; Neurotic, Net; Sensitive, Set) comprised of eight specific factors (deviant, dissocial, apathy, anxiety, inferiority, stereotyped, suspicious and paranoid).

It is said that personality traits are often related to psychopathology [9]. In the case of psychosis, there appears to be a relationship between schizotypal personality traits and schizophrenia spectrum disorders [10-12]. Susceptibility to schizophrenia occurs across a spectrum, with schizotypal personality disorder as the prototypical subsyndromal manifestation of more severe psychosis. Schizotypal personality disorder appears to share common genetic and biologic substrates with schizophrenia [13]. The latent factors that emerge when schizotypal personality traits are examined resemble the classic positive, negative and disorganized symptom dimensions of schizophrenia [12]. It also argued that schizotypal personality traits are qualitatively similar to the characteristic symptoms of schizophrenia, albeit quantitatively less severe [14]. The expression of schizotypal personality traits ranges from relatively benign perceptual experiences associated with odd beliefs to severe symptoms associated with significant psychosocial impairment, or schizotypal personality disorder [15], which can be a premorbid condition that serves as a risk factor for the development of schizophrenia [16]. Psychometrically measured schizotypy has been found to be elevated among schizophrenia patients [17] and their relatives [18], and those with higher schizotypal scores are more likely to suffer from schizophrenia [19,20]. Battaglia et al. [21] and Siever et al. [22] provide further evidence that people with higher levels of schizotypal personalities have a higher risk of developing schizophrenia as compared to those who do not. Indicators of cerebral dysfunction observed in schizophrenia spectrum disorders [11,23], including cognitive impairment and sensory gating deficits [24,25], are correlated with schizotypal traits in psychiatrically healthy individuals [26,27], which further underscores the link between schizotypal traits and schizophrenia spectrum disorders [28-30].

Recruits for such as military, police, and civil servant with schizotypal personality disorder are of particular interest because the postpubertal period is a critical one for the development of a DSM axis I disorder, with this period being associated with neuromaturational processes that can trigger the expression of latent vulnerabilities [31,32]. It is likely that some schizotypal adolescents will remain stable over time while another subgroup will develop schizophrenia. Genetic and environmental factors contribute to the stability of schizotypal traits in adolescence, with such studies using a measure designed to assess traits during this developmental period [33,34]. In order to reduce the incidence of mental disorders amongst members of large personnel recruitment units such as the military and police, such agencies tend to screen out schizotypal individuals due to increased risk of developing schizophrenia [8]. As such, it behooves researchers to understand the population distribution of such traits, including the specific spatial distributions of these traits across different regions. Such an understanding can be combined with psychological testing to help an organization such as the military to determine which regions are more likely to yield large numbers of appropriate recruits and to focus recruitment efforts on these regions accordingly.

The Psychological Selection Systems for Chinese Recruits (PSSCR) is a mental health screening system that includes a series of intelligence and personality tests used to examine the psychological suitability of potential new recruits or employees. The PSSCR is widely used when large numbers of young employees must be recruited, and thus has military, police, and civil service applications. The schizotypal personality portion of the PSSCR is a self-report questionnaire that together assesses three dimensions with eight factors [8], according to the Chinese cultural conceptualization of schizotypal traits described earlier. Self-report measures have been found to aid in the detection of schizotypal individuals in the adult population [14,35]. The clinical schizotypal personality subtests included in the PSSCR provide a useful screen of both these personality traits and schizophrenia [36,37] and has been used in this capacity since 2006.

In sum, schizotypal personality is an important risk factor for the development of schizophrenia that is currently a major public health focus. Clarifying the geographical distribution of schizotypal personality characteristics would potentially help to discriminate the spatial distribution of schizophrenia prone individuals in different districts, which (as described above) would be information of use for personal selection purposes. Such traits are also of interest to military recruiters who must select appropriate candidates [38]. Consistent with this, the aim of the present study was to investigate the spatial distribution characteristics of schizotypal personality features in Sichuan male youth. Geographical information systems (or GIS) can provide methods that have the potential to facilitate the development of schizotypal personality indices at the local level in cities or regional areas [39-42], not only for research purposes but also for the evaluation of new draft and allocation policy initiatives intended to aid recruitment of mental health employees. This study first adopted broader uses of GIS methods, focusing particularly on exploring the spatial distribution of schizotypal personality scores across Sichuan province. Geographical information systems pertaining to schizotypal personality traits of Chinese male youth recruits were built using ArcGIS 8.3 software [43], with spatial distribution maps then being made using a spatial location interpolation technique.

## Methods

### Study area

The present study area was the main island of Sichuan province, which is located at north latitude 26°03′-34°19′and east longitude 97°21′-108°31′ in southwest China. The region has an area of 486,000 square kilometers and a population of around 80.41 million individuals. Fifty-five minority groups live within province, including significant numbers of Tibetans, Yi, Qiang and Naxi residing in the western portion and forming a traditional transition zone between Central and East Asian cultures. Sichuan was China's most populous province before Chongqing was carved out of it; it is currently the fourth most populous after Guangdong, Shandong, and Henan. Sichuan is a very important province for military recruitment given that China requires compulsory military service. Around 40,000 military personnel are recruited from Sichuan every year, and one soldier in ten hails from Sichuan province.

### Data collection and management

PSSCR data collected in Sichuan in 2011 were analyzed. Data from all 67,558 male recruits screened for schizotypal personality traits in Sichuan were included. Recruit ages ranged from 18 to 25 years with mean of 22.18. The schizotypal personality measurement subscale of the PSSCR is a self-report scale comprised of 207 items. The measure includes three clinical subscales – dissociative (Dit), neurotic (Net), and sensitive (Set) – and three validity questionnaires – defensive (D), Deviant responding (DS), and truth-concealing (TC). T value for each scale can be used as predictors for schizotypal personality, and the higher the T scores of a clinical subscale, the more vulnerability to schizophrenia. This schizotypal personality test has been found to have good reliability and validity, such that schizotypal personality individuals had significantly higher scores on the clinical scales compared with those who do not have this vulnerability [8,36,37,44]. Data accuracy was ensured by national and provincial quality control teams, which reported response rates and validity measures for all recruits.

Populations of each county in 2011 were derived from the Sichuan statistical yearbooks, which were compiled by the Sichuan Provincial Bureau of Statistics. Years of education and illiteracy rates was used to explore the spatial distribution of demographic data which were collected from annual statistical reports from each county. Illiteracy rate was defined as the proportion of illiterate people in the populations who can neither read nor write at a level above school age (12–15 years old). All three subscales scores of schizotypal personality, illiteracy rate, and years of education for each county were geo-coded and attached to the corresponding polygon on a digital map of Sichuan. Parameter estimates were then performed via spatial interpolation using the ordinary kriging method, which was a common method used to infer values of a variable of interest at an unobserved location. Demographic and psychological test data were then extracted using the spatial analyst model using ArcGIS 8.3 software, by overlapping the vector county map of Sichuan on the raster maps [43].

### Geographical information system construction

The means of years of education and illiteracy rate, T-scores for the Dit, Set and Net schizotypy subscales were used to build geographical information systems for each subscale. Based on the electronic map of the county borderline of Sichuan (proportion of 1:1,000,000), Dit, Set, and Net attribute databases were constructed, and corresponding geographical information systems (GIS) for Dit, Set and Net were constructed using ArcGIS8.3 software.

### Geostatistical analysis

ArcGIS 8.3 software was used to analyze geostatistics and draw schizotypal personality spatial distribution maps of each clinical scale via kriging interpolation. Spatial patterns for schizotypal personality features were determined using geostatistical analysis, according to the every GIS.

Semivariograms were developed to evaluate the degree of spatial continuity of schizotypal personality among data points and to establish a range of spatial dependence for Dit, Net, and Set separately by using a 125 km sampling interval. Information generated through variograms was used to calculate sample-weighted factors for spatial interpolation using a kriging procedure [45]. Kriging is a linear interpolation procedure that provides a best linear unbiased estimation for quantities that vary in space [46]. The exponential model, Gaussian model and Spherical model were used to fit the experimental semivariogram. The fitted model provides information about the spatial structure as well as the input parameters such as nugget, sill and range for kriging interpolation. By fitting the appropriate variogram model, the distance dependent coefficients can be estimated and graphically interpreted.

Cross-validation was used to evaluate if each map provides optimal unbiased predictions. The following four indices of prediction error were used to evaluate optimal unbiased prediction: (a) mean prediction error (MPE) that reflects estimation bias, with the closer to 0 the better, (b) root mean-square prediction error (RMS-PE) and (c) average standard error (ASE-PE), reflecting the consistency of observed data estimation, with all such values as small as possible and as close in value as possible, and (d) root mean-square standardized predicted error (RMSS-PE), reflecting variation in the degree of prediction error; values closer to 1 reflect smaller variation.

## Results

### Spatial distribution of schizotypal personality features in Sichuan province

**Figure 1:**
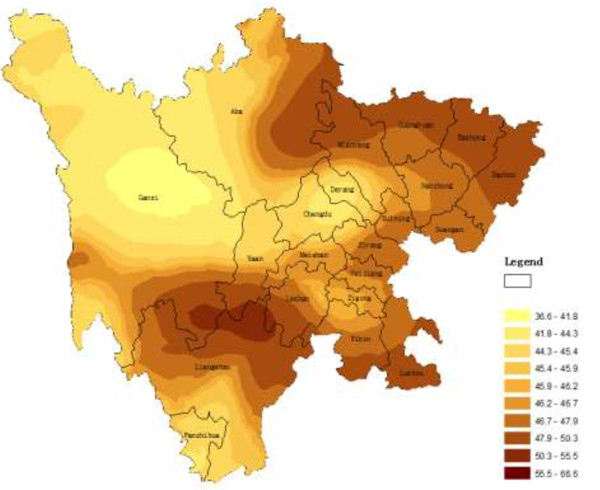
**Spatial distribution map of Dit produced by ordinary kriging.** Legend: Map colors reflect the distribution trends of T-scores of Dit in Sichuan, such that the darker the color, the greater the index value; and same color showed the same trends in different place. Similarly hereinafter.

**Figure 2:**
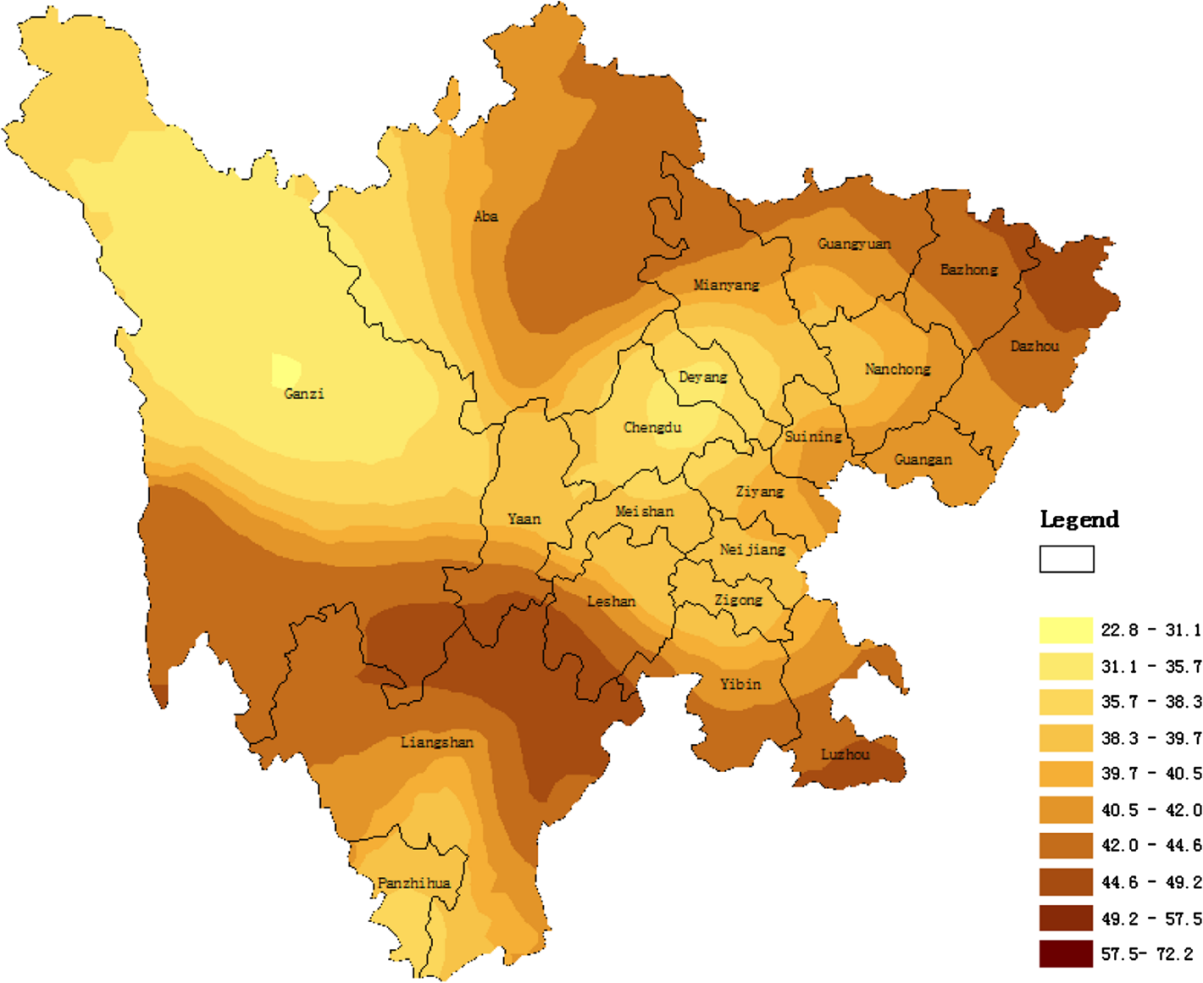
Spatial distribution map of Set produced by ordinary kriging.

**Figure 3:**
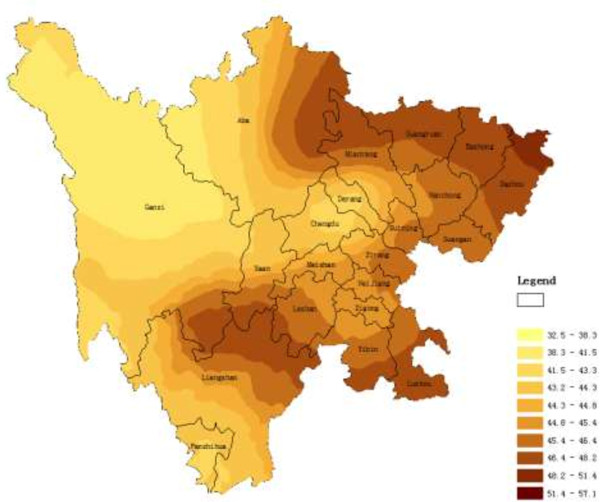
Spatial distribution map of Net produced by ordinary kriging.

The main application of geostatistics to health science has been the estimation and mapping of health attributes in nonsampled areas. Figures 1, 2, and 3 shows the spatial maps of Dit, Set and Net scores generated from corresponding semivariagrams. Map colors reflect the distribution trends of years of education in Sichuan, such that the darker the color, the greater the index value (similarly hereinafter). All the maps of the three clinical subscales showed similar geographical trends, with t-scores generally increasing from west to east, with t-scores of eastern counties generally higher than those from western counties. The highest clinical scale t-scores were distributed mainly in the eastern and northern counties of the province such as Dazhou, Bazhong, Baisha, and Guangyuan. However, high scores did cluster in certain southern and western counties such as Luzhou, Yibin, and Liangshan.

### Spatial distribution of education levels in Sichuan province

**Figure 4:**
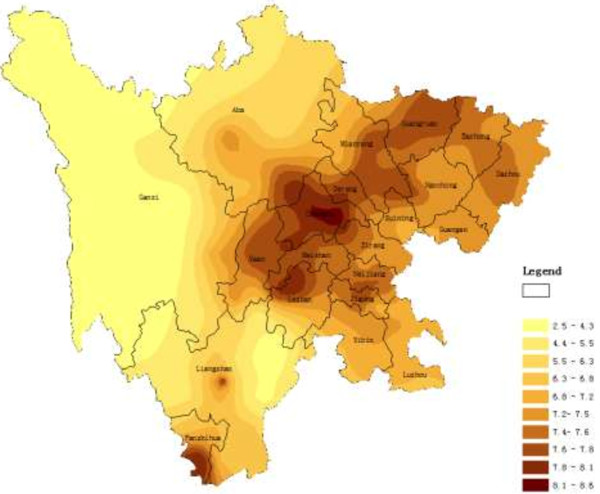
Spatial distribution map of years of education produced by ordinary kriging.

The spatial distribution map of years of education produced by ordinary kriging is shown in Figure 4. This map indicates clear regional differences in education level. The central areas of Sichuan province have a higher education level than more peripheral regions, with Chengdu having the highest education level.

**Figure 5:**
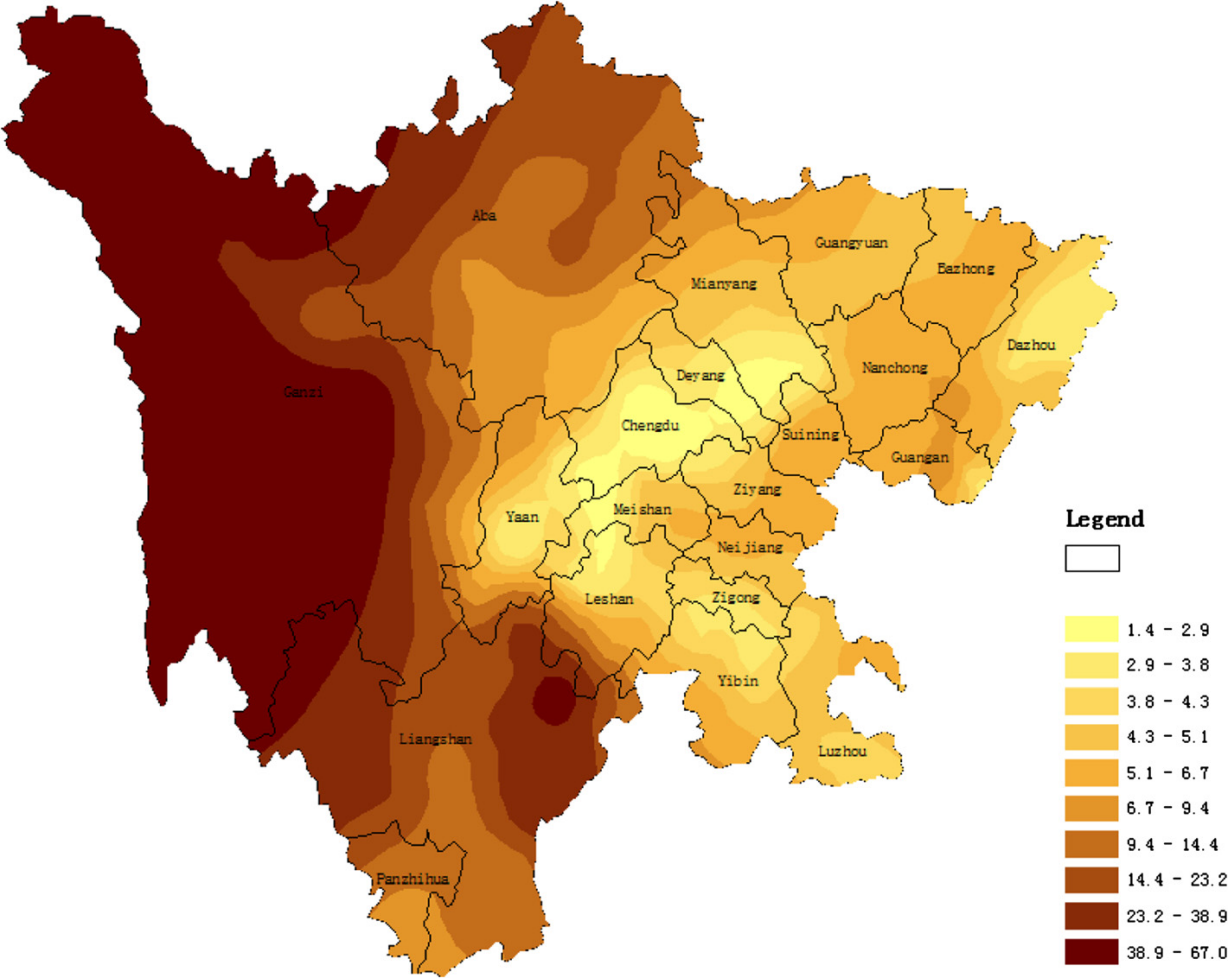
Spatial distribution map of illiteracy rate produced by ordinary kriging.

The spatial distribution map of illiteracy rate produced by ordinary kriging is shown in Figure 5. As is the case for the map of education level, color reflects the distribution trends for illiteracy rates with darker colors indicative of greater illiteracy. Again, clear regional imbalances are apparent in Sichuan province, such that central regions had lower illiteracy rates relative to more peripheral regions.

### Cross-validation of the distribution maps

Spatial distribution maps of each index were evaluated according to prediction error. From Table 1, it is apparent that MPEs for each map were relatively low and relatively close to 0, while RMSS-PE values were close to 1, indicative of relatively low levels of prediction error for each map. RMS-PE values for each map were close to corresponding ASE-PE values, suggesting that the predicted degree of variation can be properly evaluated using standard prediction error values with proper fit.

**Table 1:** **Cross**-**validation indexes for schizotypal personality map**

	**MPE**	**RMSS**-**PE**	**RMS**-**PE**	**ASE**-**PE**
Set	-0.001552	1.112	3.449	3.045
Net	0.008977	1.286	3.599	2.704
Dit	-0.002059	1.115	3.764	3.319
Years of Education	0.006951	1.074	0.6012	0.5397
Illiteracy Rate	-0.007265	1.246	7.761	5.869

### The relationship between schizotypal personality and education level

Correlational analyses were conducted to clarify the effects of demographic variables on schizotypal personality. As shown in Table 2, Set t-scores were negatively correlated with years of education. Net scores were negatively correlated with illiteracy rate; it seems odds that illiteracy going down as schizotypy goes up. These patterns are quite evident when spatial distribution maps are examined, such that negative correlations between pairs of characteristics show significant contrast when the colors are compared. Taking Set scores and years of education as an example, these maps show color patterns that approximate mirror images of one another. No other correlation coefficients were significant.

**Table 2:** Correlations between schizotypal personality scores and demographic variables

	**Set**	**Dit**	**Net**
Years of Education	-0.22**	-0.07	0.07
Illiteracy Rate	0.11	-0.02	-0.17*

*p < 0.05, ** *p* < 0.01.

## Discussion

The present study investigated the spatial distribution of schizotypal personality traits in Chinese male youth recruits in Sichaun province. Demographic variables including years of education and illiteracy rates were also spatially mapped and effects of these variables on schizotypal personality scores were examined. High schizotypal personality scores did tend to cluster in certain regions of the province, suggesting that the incidence of these traits does vary as a function of region. This finding is consistent with previous studies that investigated genetic and environmental influences on the symptom dimensions of schizotypal personality [14,35,47], although relatively few such studies have been conducted. One adult twin study suggested a latent common schizotypy factor in both males and females [48]. Behavioral genetic studies have demonstrated that heritable factors are important in the development of schizotypal personality in adults [47]. Lin et al. [35] investigated the genetic and environmental etiology of schizotypal traits during adolescence. Marissa Ericson [14] confirmed that genetic and non-shared environmental factors play an important role in the development of schizotypal traits in adolescents through a longitudinal study. So far as the present authors know, the present study was the first to examine the spatial distribution of schizotypal personality traits using the kriging method based on GIS, and the first to suggest that such traits are differentially distributed across different regions. Genetic and environmental effects likely play a role in such regional differences, meaning that these developmental factors likely show regional distribution differences as well.

Another interesting finding of the present study was that certain dimensions of schizotypal personality are significantly correlated with demographic variable such as years of education and illiteracy rate. Although no causal relationship can be inferred from the present data, schizotypal personality features do seem to be associated with regional demographic differences. The relationship between years of education and Set scores is consistent with findings from previous studies. Miettunen et al. [49] observed that higher education is associated with reduced negative schizotypy on the Social and Physical Anhedonia scales, with less educated participants scoring higher on both scales. Ma et al. [50] found an inverse relationship between years of education and scores on the No Close Friends subscale in Chinese participants. However, the negative correlation between illiteracy rate and Net scores that we observed is inconsistent with past results. Studies conducted by Guo et al. [51] and Crow et al. [52] provide positive evidence of educational problems long before illness onset. It has been suggested that low educational attainment is associated with the same deficits that drive poor social functioning in these personalities [51]. Schizotypy appears to be more common in eastern areas that are more prosperous and that have higher educational attainment. It seems erroneous to argue that low education is a protective factor based on earlier work and logic, so it would appear that schizotypy rates are lower in these western areas, which also happen to be less well educated, with no meaningful relationship between schizotypy and illiteracy at all (i.e., the negative correlation does not reflect a true causal link between these variables but is instead a by-product of the relationship between schizotypy and geography). Confusingly though, the negative correlation between these traits and years of education is consistent with earlier work and makes logical sense.

Given that schizotypal personality tends to be stable over time, with a proportion of schizotypal adolescents developing schizophrenia under certain genetic and environmental conditions [20,23,30,53,54], it could be concluded that areas with a higher incidence of schizotypal personalities should also show higher rates of schizophrenia [33,39,41,55]. These findings suggest that public health resources, particularly those concerning mental health, might be most beneficially focused on areas where incidence of schizotypal traits is greater, such that the incidence of schizophrenia might be decreased if individuals do not progress to the more severe condition.

According to the Chinese military epidemiological investigation of mental disease conducted in 1994 [56], there are more than 10,000 new recruits with various mental disorders every year, with around 162 individuals with schizophrenia for every 100,000 new recruits. Xiao et al. [8] found that schizophrenia accounted for 73% of severe mental illnesses diagnosed in a sample of 13,000 military inpatients. Such studies suggest that schizophrenia is an important health concern in the military, worthy of resources devoted to treatment and prevention. Screening out individuals with schizotypal personality disorder may constitute one approach to reduce rates of schizophrenia in the military, which should lead to improvements in overall rates of mental illness in the military. China practices compulsory military service and different counties have different recruit quotas. Such regions are subject to more rigorous screening procedures such that appropriate recruits would not be missed according to spatial distribution maps of schizotypal personality prevalence combined with further indices of education level, so as to recruit fewer youth who are at high risk of developing schizophrenia, with fewer recruits being obtained from high risk areas. Other government bodies such as the police and civil service could adopt a similar approach.

The present study does have some limitations. This study provided a useful demonstration of how to assess geographical prevalence of a mental health variable such that regions can be compared, and had potential personnel screening applications. However, one can conclude little or nothing about the potential causal relationships between schizotypal personality and geographical location/demographic variables. It would seem likely that geographical location is a proxy variable for the genuine causal factors at play or a correlate of these variables that itself lacks explanatory power. Another limitation concerns the specialized nature of the sample, which consisted of young male military recruits. Although the present maps may be useful for other applications where only male personnel are being selected, most fields would require applicants of both genders, making the present results less generalizable and decreasing the external validity of the present study.

## Conclusions

Despite some limitations, this study extends our insight into the genetic and environmental mechanisms underlying schizotypal personality, perhaps, although such mechanisms are not directly identified here. The finding that schizotypal personality cases tend to cluster in certain geographic regions could inform allocation of public health resources as well as personnel selection procedures intended to decrease mental illness rates in organizations such as the military

## Competing interests

The authors declare that they have no competing interests.

## Authors’ contributions

Conceived and designed the experiments: JZ WW ZT WX DM. Performed the experiments: JZ QU WX JP. Analyzed the data: ZT YZ LS. Contributed reagents/materials/analysis tools: WX DM YZ LS. Wrote the paper: JZ WW WX DM. All authors read and approved the final manuscript.
